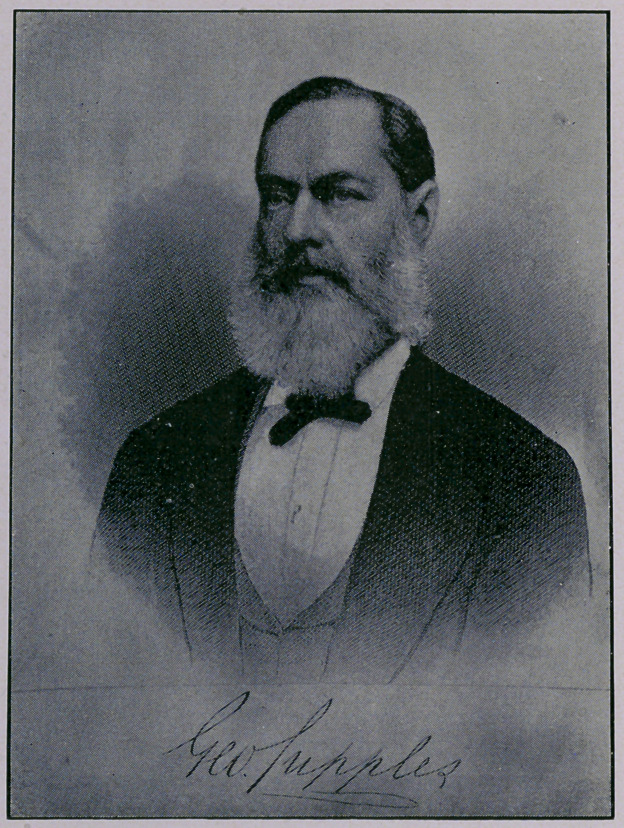# Death of Dr. Cupples

**Published:** 1895-05

**Authors:** 


### EDITORIAL DEPARTMENT.

F. E. DANIEL, M. D., Editor.
S. E. HUDSON, M. D., Managing Editor.
A. J. SMITH. M. D., Galveston, Associate Editor.

EDITORIAL STAFF:

PROF. J. E. THOMPSON, M. D., Texas Medical College, Galveston; Surgery.
PROF. WM. KEILLER, M. D., Texas Medical College, Galveston; Obstetrics and Gynecology.
PROF. DAVID CERNA, M. D., Texas Medical College, Galveston; Therapeutics.
PROF. A. J. SMITH, M. D., Texas Medical College, Galveston; Medicine
DR. R. H. L. BIBB, Saltillo, Mexico; Foreign Correspondent.

Official organ of the West Texas Medical Association, the Houston District Medical Association, the Austin District Medical Society, the Galveston County Medical Society, and several others

### DEATH OF DR. CUPPLES.

It is with feelings of profound sorrow and regret that the JOURNAL chronicles the death of that great and good man, DR. GEO. CUPPLES.

We grieve for the loss of a dear personal friend; the whole medical world shares with us the loss of the great surgeon and physician; the Texas profession, especially, is bereaved. He was our Nestor,—leader; pioneer in all that was good and great in medicine, and in his life and walk an exemplar of its highest and best attributes.

He was a progressive man, a man of resources and of action. Endowed with a broad intellectual capacity, as a practitioner he brought to bear a mind stored with the lore of three continents, ripened and matured by an experience of half a century. As a surgeon he had scarcely an equal; a superior, nowhere. His work is immortal. He was a rare man. Only those who were admitted into his affections really knew him; to strangers he appeared dignified to austerity, reserved even to repulsion. He was not what is called a *popular* man. But to his friends he was gentleness and goodness personified, open, frank, generous; full of quaint humor, and a boon companion in leisure hours. His rugged Scotch character had received, in early days, a French veneering, the result of student life in Paris some years, and politeness and courtesy were his characteristics; the "American," however, predominated at last. Dr. Paschal, in his beautiful oration, which we herewith reproduce from the *S. A. Express*, has paid such spendid tribute to his memory, that we are impoverished, and can find no fitting words in which to express our own sense of bereavement.

Accompanying this we reproduce his likeness from a steel engraving, made some ten years ago. Of late years he had had no picture taken. We learn that Dr. Cupples leaves much valuable matter in manuscript, which, the JOURNAL hopes, one day his friends will cause to be published.

Notwithstanding his advanged age (79 years), to the hour of his death his mind was unclouded, while his physical energy and strength were matter of common comment. His hand was as steady as at the age of twenty,—his mind as clear, his judgment as unerring. He was performing laparatomies a day or so before he was stricken with the malady which robbed us of our favorite, and the West of her great surgeon. (He is reported to have died of some intestinal obstruction.)

As exemplifying further his physical strength, he drove—he drove himself oftener than otherwise—the most spirited horses; he would have no other, and always drove a pair. The love of horses was one of his characteristics. He was denied the happiness of having children of his own, but he took to his heart with all the warmth of a father's love, little Georgie and Tod, the little boys of his step-daughter, Mrs. Lamkin.

In the bright Empyrean realms, far beyond the skies,—the abode of noble souls,—illumined and made glorious by the presence of Eternal God, may his gentle spirit find its guerdon of rest; and when the arch-angel shall recount his many benefactions to suffering man, may a crown of glory, too, be his. Amen.

### BIOGRAPHICAL.

Dr. George Cupples was of distinguished Scottish ancestry, himself and father having been born in Scotland. Dr. Cupples' father was a surgeon in the English navy, and his mother's father, John Campbell, was a captain in the English navy.

Dr. George Cupples himself became connected with naval service, having enlisted as staff assistant surgeon in the Spanish service in 1836. In 1838 he resigned, and in August of the same year he graduated from the University of Edinburgh, studying also in the hospitals of London. Dr. Cupples then went to Paris where he studied until 1843 for the purpose of occupying an official position in the hospitals of France. In 1844 he sailed for America, coming direct to San Antonio from Paris. During the Mexican war he enlisted in Hays' second regiment of cavalry and served throughout that struggle as a surgeon. When the civil war began he entered the Confederate army as first surgeon of the 7th Texas regiment of mounted volunteers, serving in the campaign of New Mexico. In December, 1862, he was appointed Medical Director of the Eastern Military District of Texas, and continued in charge until he was ordered to join Sibley's brigade in Louisiana. The following summer he served as senior chief surgeon of division, and in June, 1864, as Medical Director and Inspector of the cavalry corps of the Trans-Mississippi department up to the close of the Red River campaign. He gave his parole at San Antonio at the surrender.

Dr. Cupples was President of the Texas State Medical Association in 1874, and again in 1878. His chief distinction was won by his achievements in surgery. He was the first to introduce into Texas anesthetics—ether first and chloroform afterwards. He was the first in the United States to perform the extirpation of the tongue for cancer, by Nunnelly's method, and to perform the operation of ovariotomy in a child under eight years of age, and to perform Freunde's operation for the extirpation of uterus and ovaries. The first two operations were successful, but in the third the patient died within fifty-four hours. He was the first in Texas to amputate at the hip joint and knee joint with success.

Dr. Cupples was twice married. His first wife, whom he married in Paris, was a Miss Alexie Bourland, a Belgium lady. She became an invalid shortly after their marriage, and it was the hope of bettering her health that induced Dr. Cupples to come to San Antonio. The climate benefited her, for she lived sixteen years after moving to this city. In June, 1874, Dr. Cupples married a second time, the lady being Mrs. Laura L. Sheahan. His wife and a step-daughter (Mrs. Lamkin) survive him.

## Figures and Tables

**Figure f1:**